# Intercalated disc in failing hearts from patients with dilated cardiomyopathy: Its role in the depressed left ventricular function

**DOI:** 10.1371/journal.pone.0185062

**Published:** 2017-09-21

**Authors:** Ana Ortega, Estefanía Tarazón, Carolina Gil-Cayuela, María García-Manzanares, Luis Martínez-Dolz, Francisca Lago, José Ramón González-Juanatey, Juan Cinca, Esther Jorge, Manuel Portolés, Esther Roselló-Lletí, Miguel Rivera

**Affiliations:** 1 Cardiocirculatory Unit, Health Research Institute La Fe, Valencia, Spain; 2 Center for Biomedical Research Network in Cardiovascular Diseases (CIBERCV), Madrid, Spain; 3 Heart Failure and Transplantation Unit, Cardiology Department, University and Polytechnic La Fe Hospital, Valencia, Spain; 4 Cellular and Molecular Cardiology Research Unit, Department of Cardiology and Institute of Biomedical Research, University Clinical Hospital, Santiago de Compostela, Spain; 5 Cardiology Service of Santa Creu i Sant Pau Hospital, Barcelona, Spain; Scuola Superiore Sant'Anna, ITALY

## Abstract

Alterations in myocardial structure and reduced cardiomyocyte adhesions have been previously described in dilated cardiomyopathy (DCM). We studied the transcriptome of cell adhesion molecules in these patients and their relationships with left ventricular (LV) function decay. We also visualized the intercalated disc (ID) structure and organization. The transcriptomic profile of 23 explanted LV samples was analyzed using RNA-sequencing (13 DCM, 10 control [CNT]), focusing on cell adhesion genes. Electron microscopy analysis to visualize ID structural differences and immunohistochemistry experiments of ID proteins was also performed. RT-qPCR and western blot experiments were carried out on ID components. We found 29 differentially expressed genes, most of all, constituents of the ID structure. We found that the expression of *GJA3*, *DSP* and *CTNNA3* was directly associated with LV ejection fraction (*r* = 0.741, *P* = 0.004; *r* = 0.674, *P* = 0.011 and *r* = 0.565, *P* = 0.044, respectively), LV systolic (*P* = 0.003, *P* = 0.003, *P* = 0.028, respectively) and diastolic dimensions (*P* = 0.006, *P* = 0.001, *P* = 0.025, respectively). Electron microscopy micrographs showed a reduced ID convolution index and immunogold labeling of connexin 46 (*GJA* gene), desmoplakin (*DSP* gene) and catenin α-3 (*CTNNA3* gene) proteins in DCM patients. Moreover, we observed that protein and mRNA levels analyzed by RT-qPCR of these ID components were diminished in DCM group. In conclusion, we report significant gene and protein expression changes and found that the ID components *GJA3*, *DSP* and *CTNNA3* were highly related to LV function. Microscopic observations indicated that ID is structurally compromised in these patients. These findings give new data for understanding the ventricular depression that characterizes DCM, opening new therapeutic perspectives for these critically diseased patients.

## Introduction

Dilated cardiomyopathy (DCM), one of the most frequent causes of heart failure (HF), is characterized by ventricular dysfunction, impaired myocardial contractility, abnormal wall thickness, and cardiac chambers dilation [1, 2]. Several alterations have been associated with this disease [3, 4]. However, despite the advances in the understanding of its molecular basis, no specific treatment is still available.

Heart muscle contraction is compromised in a dilated myocardium. The intercalated disc (ID) structure includes cell adhesion molecules that form cell junctions allowing contraction coupling of cardiomyocytes, as well as ensuring the electrical and mechanical connection between cardiac fibers [5]. The ID is composed of desmosomes, adherens junctions (AJ), and gap junctions (GJ) [6]. Some studies have linked genetic alterations in desmosomal genes to cardiomyopathies; specifically, desmoplakin (*DSP* gene) mutations have been implicated in DCM [7]. AJ, the predominant junction of ID, is composed of cadherins, which are connected intracellularly to the actin cytoskeleton by catenins [8]. Alpha catenins are found at high levels in myocardial tissues and contribute to this strong cell-cell adhesion; therefore, in patients with DCM and other cardiomyopathies, mutations in these genes have been detected on screening [9]. The third component of ID structure are the GJ, composed of connexins, which mediate electrical coupling of cardiac muscle [10]. The important role of some connexins in diseased myocardium is known, such as studies analyzing connexin 43 alterations in HF [11, 12], but other connexin genes remain to be studied in DCM.

Despite previous works showing the relevance of adhesion junctions for cardiac contraction and function, we lack studies analyzing the transcriptomic profile of cell adhesion molecules in DCM, including the ID components. We hypothesized that there may be important alterations regarding cell adhesion in HF, specifically in the ID at the genetic and structural levels. Therefore, we analyzed the differentially expressed cell junction genes in DCM patients and compared them with those in control (CNT) subjects. We also calculated the relationship of these changes with left ventricular (LV) function decay and investigated the ID structural organization.

## Material and methods

### Collection of cardiac tissue samples

Twenty-three LV tissue samples were obtained from patients with DCM (*n* = 13) undergoing heart transplantation and non-diseased CNT donors (*n* = 10). DCM sample size was increased to perform RT-qPCR (*n* = 20) and western blot (*n* = 25) experiments. Patients were diagnosed based on clinical history, electrocardiogram, hemodynamic studies, Doppler echocardiography, and coronary angiography data. These patients had intact coronary arteries, as seen on coronary angiography, and LV systolic dysfunction (ejection fraction (EF) <40%) with a dilated left ventricle (LV end-diastolic diameter [LVEDD] >55 mm). Patients with primary valvular disease or familial DCM were excluded from the study. All patients were functionally classified according to the New York Heart Association (NYHA) criteria and received medical treatment in agreement with the guidelines of the European Society of Cardiology [13]. Table 1 summarizes the clinical characteristics of DCM patients.

**Table 1:** Clinical characteristics of DCM patients.

	DCM (*n* = 13)	DCM (*n* = 20)	DCM (*n* = 25)
	RNA-sequencing	RT-qPCR	Western blot
Age (years)	51 ± 11	50 ± 13	49 ± 15
Gender male (%)	92	70	76
NYHA class	3.4 ± 0.4	3.1 ± 0.7	3.4 ± 0.4
BMI (kg/m^2^)	27 ± 5	25 ± 5	25 ± 7
Hemoglobin (mg/dL)	13 ± 3	13 ± 3	13 ± 2
Hematocrit (%)	39 ± 8	39 ± 7	40 ± 5
Total cholesterol (mg/dL)	147 ± 37	136 ± 43	145 ± 47
Prior hypertension (%)	17	20	23
Prior smoking (%)	50	40	64
Diabetes mellitus (%)	17	23	17
EF (%)	20 ± 7	21 ± 10	21 ± 8
LVESD (mm)	71 ± 12	67 ± 13	66 ± 11
LVEDD (mm)	80 ± 11	74 ± 12	74 ± 11
Left ventricle mass index (g/m^2^)	241 ± 77	194 ± 54	207 ± 45
Duration of disease (months)	75 ± 68	70 ± 55	64 ± 53

DCM, dilated cardiomyopathy; NYHA, New York Heart Association; BMI, body mass index; EF, ejection fraction; LVESD, left ventricular end-systolic diameter; LVEDD, left ventricular end-diastolic diameter; Duration of the disease, from DCM diagnosis until heart transplant.

The CNT samples were hearts with normal LV function and no history of myocardial disease at the time of transplantation. These hearts were initially considered for heart transplantation, but were classified as unsuitable owing to blood type or size incompatibility. The causes of death of these CNT group were motor vehicle accidents and ictus.

The present study was approved by the Ethics Committee (Biomedical Investigation Ethics Committee of La Fe University Hospital of Valencia, Spain) and was conformed in accordance with the principles outlined in the Declaration of Helsinki [14]. All heart samples were obtained with written informed consent of the patients or their families.

Tissue samples were obtained from near the apex of the left ventricle and maintained in 0.9% NaCl at 4°C for a maximum of 4.4 ± 3 h after the coronary circulation loss, and then stored at –80°C until RNA and protein extraction. The RNA integrity number (RIN) obtained from our samples was ≥ 9, which was our intragroup condition for being included in this study. Proper handling and rapid sample collection and storage by our on-call (24 h/day) team enabled us to obtain these high-quality samples. The access to the operating room during transplantation surgery allowed us to select tissue samples from the same LV area, standardizing our research methodology. The samples were handled uniformly in both groups.

### RNA extraction

Heart tissue samples were homogenized using TRIzol® reagent in TissueLyser LT (Qiagen; Manchester, UK). RNA was extracted using the PureLink™ Kit (Ambion Life Technologies; Carlsbad; CA, USA), following the manufacturer’s instructions. RNA concentration was measured on the Nanodrop 1000 spectrophotometer (Thermo Fisher Scientific; Leicestershire, UK), and the purity and integrity of RNA samples were measured using the microfluidics-based platform 2100 Bioanalyzer with the RNA 6000 Nano LabChip Kit (Agilent Technologies; Santa Clara, CA, USA). All RNA samples displayed a 260/280 absorbance ratio ≥ 2.0 and reached a minimal RIN ≥ 9.

### RNA-sequencing analysis

Poly (A)—RNA samples were isolated from 25 μg of total RNA using the MicroPoly (A) Purist Kit (Ambion, Life Technologies, Carlsbad, CA, USA). The SOLiD 5500 XL platform (Life Technologies; Carlsbad, CA, USA) was used for sequencing whole transcriptome libraries, generated from total poly (A)—RNA samples, following the manufacturer’s recommendation. No RNA-spike was used in controls. Amplified cDNA quality was analyzed using the Bioanalyzer 2100 DNA 1000 Kit (Agilent Technologies; Santa Clara, CA, USA) and quantified using the Qubit 2.0 Fluorometer (Invitrogen; Paisley, UK). The whole transcriptome libraries were used for making SOLiD-templated beads by following the SOLiD Templated Bead Preparation guidelines. The bead quality was assessed based on the workflow analysis parameters. The samples were sequenced using the 50625 paired-end protocol generating 75 nt + 35 nt (paired-end) + 5 nt (barcode) sequences. Quality data were measured using SOLiD Experimental Tracking Software parameters.

### RNA-sequencing data computational analysis

The initial whole transcriptome paired-end reads obtained from sequencing were mapped against the latest version of the human genome (version GRchr37/hg19) by using the Life Technologies mapping algorithm (http://www.lifetechnologies.com/; version 1.3). The aligned records were reported in BAM/SAM format [15]. The Picard Tool (http://picard.sourceforge.net/; version 1.83) was used to eliminate insufficient quality reads (Phred score < 10). Subsequently, gene predictions were estimated using the Cufflinks method [16], and the expression levels were calculated by using HTSeq software [17]. This method eliminates the multi-mapped reads, so that only the unique reads are considered for gene expression estimation. The differential expression analysis between conditions was assessed by the edgeR method (version 3.2.4) [18]. This method relies on different normalization processes based on the depth of global samples, the CG composition, and the gene length. Moreover, this method is based on a Poisson model that estimates the variance of the RNA-sequencing data for differential expression. Finally, we selected genes showing differential expression at a significance threshold of *P* < 0.05. The data presented in this paper have been deposited in NCBI’s Gene Expression Omnibus (GEO) [19] and are accessible through GEO Series accession number GSE55296 (http://www.ncbi.nlm.nih.gov/geo/query/acc.cgi?acc=GSE55296).

### RT-qPCR analysis

One microgram of total RNA from 20 DCM and 10 CNT LV samples was reverse-transcribed to cDNA using the M-MLV enzyme (Invitrogen, UK). RT-qPCR was performed in duplicate using the TaqMan protocol in a ViiA7 Fast Real-Time PCR System according to the manufacturer’s instructions (Applied Biosystems; USA). The following TaqMan probes were obtained from Life Technologies: *GJA3* (Hs00254296_s1), *DSP* (Hs00950591_m1) and *CTNNA3* (Hs00379052_m1) the housekeeping genes *GAPDH* (Hs99999905_m1), *PGK1* (Hs99999906_m1), and *TFRC* (Hs00951083_m1) were used as endogenous controls. Relative gene expression levels were calculated using the 2^−ΔΔCT^ method [20].

### Gene functional enrichment

We performed a functional enrichment analysis of differentially expressed genes based on hypergeometric testing using the ToppGene suite [21]. We selected the differentially expressed genes from DCM patients with ≥ 1.3–fold and *P* < 0.05 by using the Bonferroni correction. Next, the most significant functional categories altered in DCM patients were represented.

### Homogenization of samples and protein determination

Twenty-five milligrams of frozen left ventricle were transferred into Lysing Matrix D tubes designed for the FastPrep-24 homogenizer (MP Biomedicals, USA) in a total protein extraction buffer (2% SDS, 10 mM EDTA, 6 mM Tris–HCl, pH 7.4) with protease inhibitors (25 μg/mL aprotinin and 10 μg/mL leupeptin). The homogenates were centrifuged and supernatant aliquoted. The protein content of the aliquot was determined using Peterson’s modification of the micro Lowry method with bovine serum albumin (BSA) as the standard.

### Polyacrylamide gel electrophoresis and western blot analysis

Protein samples for the detection of desmoplakin were separated using Tris-Acetate Midi gel electrophoresis with 3–8% polyacrylamide. Bis-Tris Midi gel electrophoresis with 4–12% polyacrylamide was used for the detection of catenin α-3 and connexin 46 proteins. After electrophoresis, the proteins were transferred from the gel to a PVDF membrane using the iBlot Dry Blotting System (Invitrogen Ltd, UK) for western blot analysis. The membranes were blocked overnight at 4°C with 1% BSA in Tris buffer solution containing 0.05% Tween 20 and, after blocking, were incubated for 2 h with primary antibody in the same buffer. The following antibodies were used: anti-GJA3 (connexin 46) rabbit polyclonal (PA-5 11634, 1/1000) from Thermo Fisher Scientific, anti-desmoplakin rabbit monoclonal (ab109445, 1/1000), anti-CTNNA3 (catenin α-3) rabbit monoclonal (ab184916, 1/5000) and anti-GAPDH (loading control) mouse monoclonal, (ab9484, 1/1000) from Abcam. The bands were visualized using an acid phosphatase-conjugated secondary antibody and nitro blue tetrazolium/5-bromo-4-chloro-3-indolyl phosphate (NBT/BCIP, Sigma-Aldrich, St. Louis, USA) substrate system. Finally, the membranes were digitalized using an image analyzer (DNR Bio-Imagining Systems, Israel) and quantified with the GelQuant Pro (v. 12.2) program.

### Immunohistochemistry and transmission electron microscopy

LV samples (size 1 mm3) were fixed in a solution of 1.5% glutaraldehyde and 1% formaldehyde in a 0.05 M cacodylate buffer (pH 7.4) for 1 h at 4°C. Later, the samples were post-fixed in buffered 1% OsO4 for 1 h at 4°C, dehydrated in a series of ethanol solutions, and embedded in Epon 812. Semi-thin sections were first evaluated with a light microscope (Olympus BX-50) before proceeding to evaluating ultra-thin sections (Ultramicrotome Leica EM UC6). These sections (80 nm) were obtained and mounted on nickel and cooper grids and counter-stained with 2% uranyl acetate for 20 min and 2.7% lead citrate for 3 min.

For immunogold labeling ultra-thin sections were initially incubated on sodium metaperiodate (S1878) from Sigma [22], for 2 h in a moist chamber at RT. After rinses with bi-distilled water, sections were incubated for 5 min with 3% hydrogen peroxide. Then, were floated on 0.1% BSA-Tris buffer (20 mM Tris- HCl, 0.9% NaCl, pH 7.4, containing 0.1% BSA, type V) supplemented with 5% inactivated FCS for 30 min at 37°C in a moist chamber. The grids were rinsed again with bi-distilled water and incubated in a moist chamber overnight at RT with a primary antibody diluted in 0.1% BSA-Tris buffer supplemented with 1% inactivated FCS. Anti-GJA3 (connexin 46) rabbit polyclonal (PA-5 11634, 1/100) from Thermo Fisher Scientific, anti-desmoplakin rabbit monoclonal (ab109445, 1/250), anti-CTNNA3 (catenin α-3) rabbit monoclonal (ab184916, 1/1000) from Abcam were used as primary detection antibodies separately. After rinses with 0.1% BSA-Tris buffer, the sections were incubated in a moist chamber for 1 h at 37°C with anti-rabbit IgG-gold antibody (10 nm, G3779, 1/10) from Sigma diluted in 0.1% BSA-Tris buffer containing 0.05% Tween-20 and supplemented with 5% inactivated FCS. After rinses with 0.1% BSA-Tris buffer and bi-distilled water, the sections were air dried and counterstained, first with uranyl acetate for 20 min and then with lead citrate for 5 sec.

Finally, the grids were air dried completely and examined under a JEOL JEM-1010 system (Massachusetts, USA), with magnifications ranging from X3000–12000. The micrographs were obtained by a successive selection of sections using systematic uniform random sampling [23]. Before analyzing the electron microphotographs, the tissue sections were inspected to avoid contraction bands and artefactual changes. For the histomorphometric analysis of the ID, the procedure described by Basso et al. [24] was used. The convolution index was expressed as the real distance of the ID divided by the end-to-end distance.

### Statistical methods

Data were expressed as the mean ± standard deviation for continuous variables and as percentage values for discrete variables. The Kolmogorov-Smirnov test was applied for analyzing the data distribution. Clinical characteristics of patients were compared by using Student’s t-test for continuous variables and Fisher’s exact test for discrete variables. Significant mean differences in the mRNA and protein levels between groups with a normal distribution were analyzed by using Student’s t-test, and the nonparametric Mann-Whitney *U* test was performed for comparisons between data that were non-normally-distributed. The *VCAM* mRNA levels exhibited a non-normal distribution, were log transformed (and proved to be normalized) before parametric correlation analysis. Finally, Pearson’s correlation coefficients were calculated to determine the relationships among variables and between variables and the echocardiographic parameters. *P* < 0.05 was considered statistically significant. All statistical analysis was performed using the SPSS software (version 20.0) for Windows (IBM SPSS Inc. Chicago. IL, USA).

## Results

### Clinical characteristics of DCM patients

We analyzed 23 LV tissue samples from 13 patients with DCM undergoing heart transplantation, and 10 hearts from non-diseased CNT donors for RNA-sequencing analysis, increasing the sample size up to 20 and 25 DCM samples for RT-qPCR and for western blot experiments, respectively. A majority of the patients were men (92%, 70% and 76%, respectively), and were previously classified with an NYHA functional classification of III–IV and diagnosed with significant comorbidities, including hypertension and diabetes mellitus. Table 1 summarizes the clinical characteristics of the patients included in the study.

The CNT group was also mainly composed of men (80%), with a similar mean age of 47 ± 16 years. Comorbidities and other echocardiographic data were not available for the CNT group, in accordance with the Spanish Organic Law on Data Protection 15/1999.

### RNA-sequencing analysis and functional categories enrichment

We carried out a transcriptomic analysis using RNA-sequencing to identify differentially expressed genes between the DCM and CNT groups. We found 2398 genes altered between both groups (≥ 1.3–fold, *P* < 0.05), of which 935 were up-regulated and 1463 were down-regulated.

**Fig 1:**
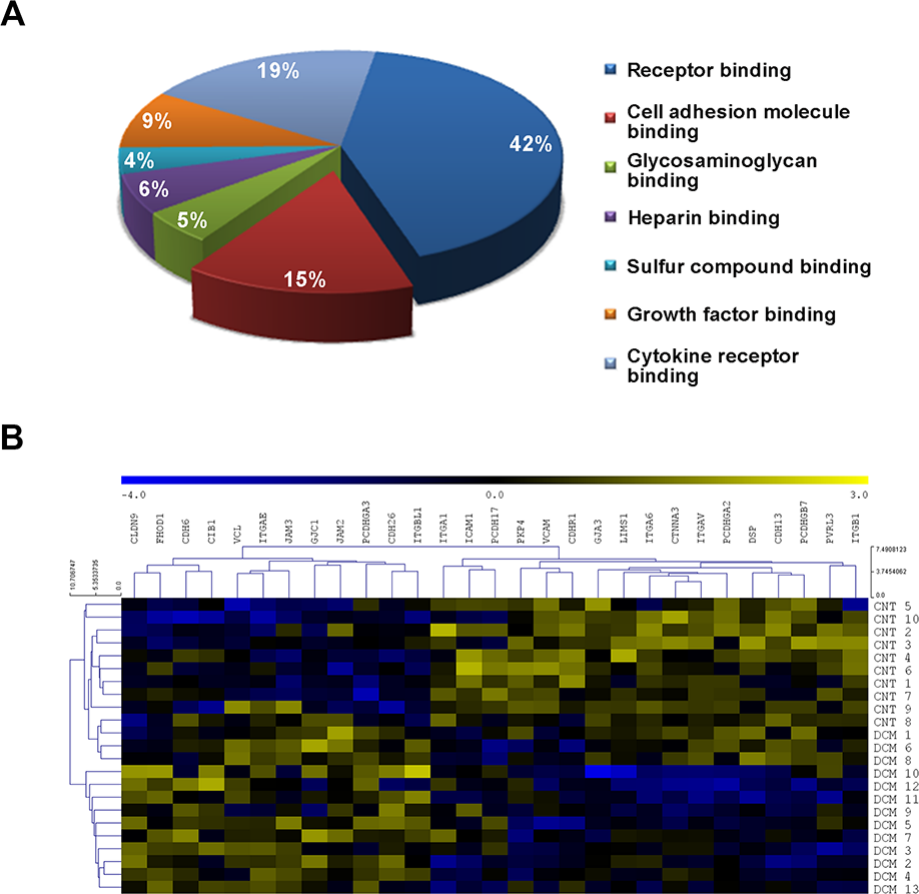
Functional classification of differentially expressed genes and transcriptomic differences visualization of cell adhesion molecules in DCM patients. **(A)** Gene functional characterization showing that cell adhesion is the third most altered category in DCM pathology. ToppGene results in terms of Gene ontology (GO) were obtained based on Molecular Function and were corrected using the Bonferroni method. The diagram includes the main categories in which differentially expressed genes are classified. **(B)** Heat map with hierarchical clustering displaying the differential profile of the 29 altered genes between the DCM and CNT groups. Columns: genes; rows: samples. The relative expression level of each gene is indicated by the color bar: blue, lowest; yellow, highest. CNT, control; DCM, dilated cardiomyopathy.

Next, we performed a functional enrichment analysis, by using the ToppGene suite tool, to establish the main biological categories in which the deregulated genes are included. We analyzed the Gene Ontology (GO) terms in the “Molecular Function” classification and we found that the third most relevant functional category was related to cell adhesion, representing 15% of the total categories (Fig 1A).

Focusing our analysis on cell adhesion molecules (S1 Table), we found 29 differentially expressed genes between the DCM patients and the CNT group (≥ 1.3–fold, *P* < 0.05) (Table 2), of which 41% are components of the ID cardiac structure. We performed a hierarchical clustering and a heat map analysis to visualize the transcriptomic differences between the DCM and CNT groups, clearly identifying the two groups of study and revealing the existence of two separate gene expression profiles (Fig 1B).

**Table 2:** Differentially expressed genes of adhesion molecules in DCM patients.

Gene symbol	Description	Fold Change	*P*-value
*CDH6*	Cadherin 6, type 2, K-cadherin	1.44	0.032
*CDH13*	Cadherin 13, H-cadherin	-1.64	0.011
*CDH26*	Cadherin-like protein 26	2.47	0.002
*CDHR1*	Cadherin-related family member 1	-2.13	0.004
*CIB1*	Calcium and integrin-binding protein 1	1.30	0.018
*CLDN9*	Claudin 9	2.78	0.003
*CTNNA3*	Catenin alpha 3	-1.49	0.021
*DSP*	Desmoplakin	-1.30	0.028
*FHOD1*	FH1/FH2 domain-containing protein 1	1.71	0.012
*GJA3*	GAP junction alpha protein 3, connexin 46	-1.62	5.4 x 10^−3^
*GJC1*	GAP junction gamma protein 1, connexin 45	1.30	0.027
*ICAM1*	Intercellular adhesion molecule 1	-2.46	0.036
*ITGA1*	Integrin, alpha 1	-1.33	0.009
*ITGA6*	Integrin, alpha 6	-1.78	0.004
*ITGAE*	Integrin, alpha E	1.54	0.003
*ITGAV*	Integrin, alpha V	-1.52	0.012
*ITGB1*	Integrin beta 1	-1.51	0.007
*ITGBL1*	Integrin beta-like protein 1	1.83	0.023
*JAM2*	Junctional adhesion molecule B	1.30	0.034
*JAM3*	Junctional adhesion molecule C	1.30	0.018
*LIMS1*	LIM and senescent cell antigen-like-containing domain protein 1	-1.36	0.014
*PCDH17*	Protocadherin 17	-1.35	0.036
*PCDHGA2*	Protocadherin gamma subfamily A, 2	-1.62	0.014
*PCDHGA3*	Protocadherin gamma subfamily A, 3	2.00	2.8 x 10^−4^
*PCDHGB7*	Protocadherin gamma subfamily B, 7	-1.48	0.020
*PKP4*	Plakophilin 4	-1.34	4.7 x 10^−4^
*PVRL3*	Nectin 3	-1.49	0.025
*VCL*	Vinculin	1.35	0.031
*VCAM1*	Vascular cell adhesion molecule 1	-3.32	0.006

### Relationships with LV function and between adhesion genes

We investigated the relationships between the differentially expressed genes, coding for cell adhesion molecules, and LV function. The functional parameters of all DCM patients were completely available.

**Fig 2:**
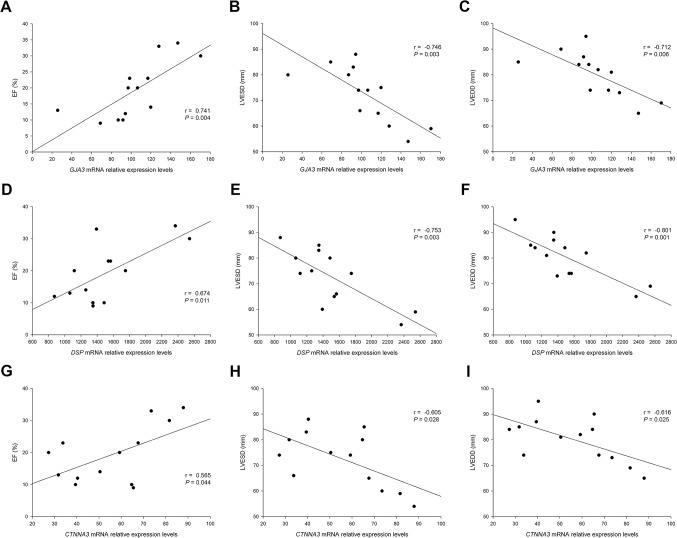
Relationships of ID genes to EF, LVESD, and LVEDD of DCM patients. **(A–C)** Simple correlations of the *GJA3* gene. **(D–F)** Simple correlations of the *DSP* gene. **(G–I)** Simple correlations of the *CTNNA3* gene. ID, intercalated disc; EF, ejection fraction; LVESD, left ventricular end-systolic diameter; LVEDD, left ventricular end-diastolic diameter; DCM, dilated cardiomyopathy.

We found that the connexin gene *GJA3*, the desmosomal gene *DSP* and the catenin gene *CTNNA3* were all significantly and directly related to the EF (*r* = 0.741, *P* = 0.004; *r* = 0.674, *P* = 0.011 and *r* = 0.565, *P* = 0.044, respectively) (Fig 2A, 2D and 2G). With regard to the ventricular morphology marked by the compensatory remodeling, both *GJA3*, *DSP* and *CTNNA3*, have high relationships with LV end-systolic diameter (*r* = -0.746, *P* = 0.003; *r* = -0.753, *P* = 0.003 and *r* = -0.605, *P* = 0.028, respectively) (Fig 2B, 2E and 2H).and LVEDD (*r* = -0.712, *P* = 0.006, *r* = -0.801, *P* = 0.001 and *r* = -0.616, *P* = 0.025, respectively) (Fig 2C, 2F and 2I).

**Fig 3:**
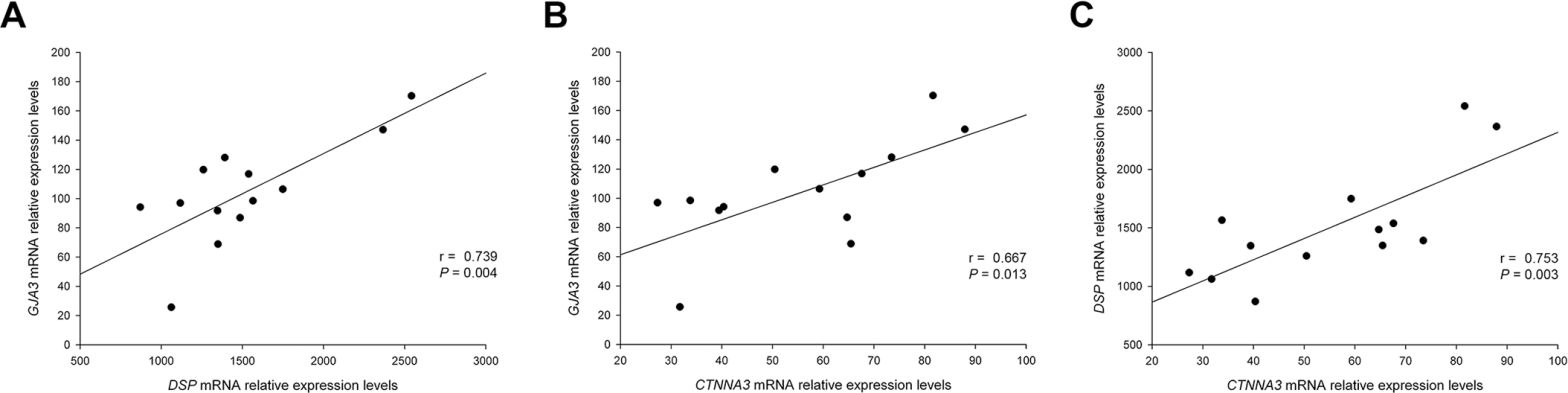
Relationships of ID genes related to LV dysfunction between them in DCM patients. **(A)** Simple correlation between *GJA3* and *DSP* genes. **(B)** Simple correlation between *GJA3* and *CTNNA3* genes. **(C)** Simple correlation between *DSP* and *CTNNA3* genes. ID, intercalated disc; LV, left ventricular; DCM, dilated cardiomyopathy.

We further analyzed the relationships of the genes linked to LV function and we found that all three genes were also related between them. *GJA3* showed relationships with *DSP* (*r* = 0.739, *P* = 0.004) and with *CTNNA3* (*r* = 0.667, *P* = 0.013) and *DSP* showed a relationship with *CTNNA3* (*r* = 0.753, *P* = 0.003) (Fig 3).

### RT-qPCR analysis and western blot experiments

**Fig 4:**
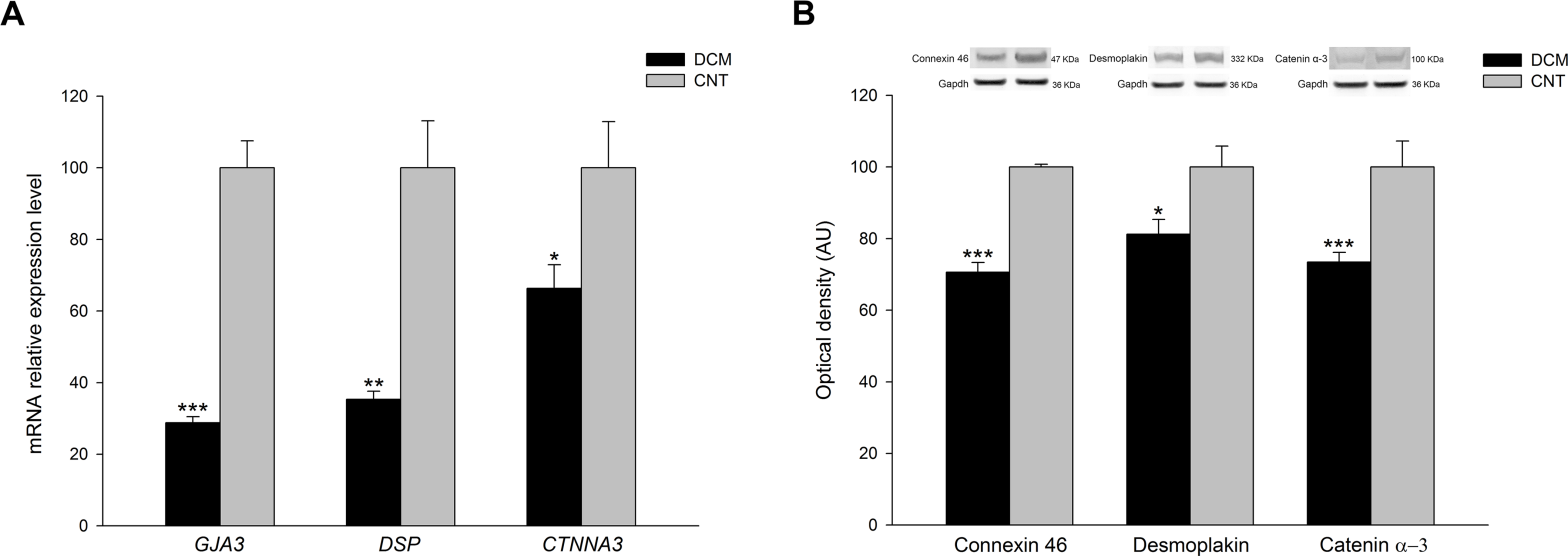
RT-qPCR validation of reduced mRNA expression and protein levels of connexin 46, desmoplakin and catenin α-3 in DCM patients. **(A)** Bar graphs representing the mRNA relative expression levels of the three genes in DCM and CNT samples. **(B)** Bar graphs indicating the relative quantification of the three proteins in DCM and CNT samples. The values of the CNT group were set to 100, and were previously normalized to three housekeeping genes in RT-qPCR analysis and to GAPDH in western blot experiments. The data are expressed as mean ± standard error of the mean of the relative mRNA expression levels and in optical density AU in the protein levels. *P < 0.05, **P < 0.01, ***P < 0.001 *vs*. the CNT group. AU, arbitrary units; DCM, dilated cardiomyopathy; CNT, control.

We performed RT-qPCR analyses of the three ID genes related to functional parameters to validate the RNA-sequencing results and increase the previous sample size. We found that *GJA3* (-3.47 fold change, *P* < 0.001), *DSP* (-2.83 fold change, *P* < 0.01) and *CTNNA3* (-1.51 fold change, *P* < 0.05) genes were all downregulated in DCM patients compared to CNT subjects (Fig 4A).

In addition, we carried out protein quantifications of these molecules and we found that protein levels of connexin 46 (71 ± 13 *vs*. 100 ± 18, *P* < 0.001), desmoplakin (81 ± 21 *vs*. 100 ± 15, *P* < 0.05) and catenin α-3 (73 ± 13 *vs*. 100 ± 22, *P* < 0.001) were all reduced respect to CNT samples, in accordance with the previously measured mRNA levels (Fig 4B).

### Intercalated disc structure and immunohistochemistry analysis

**Fig 5:**
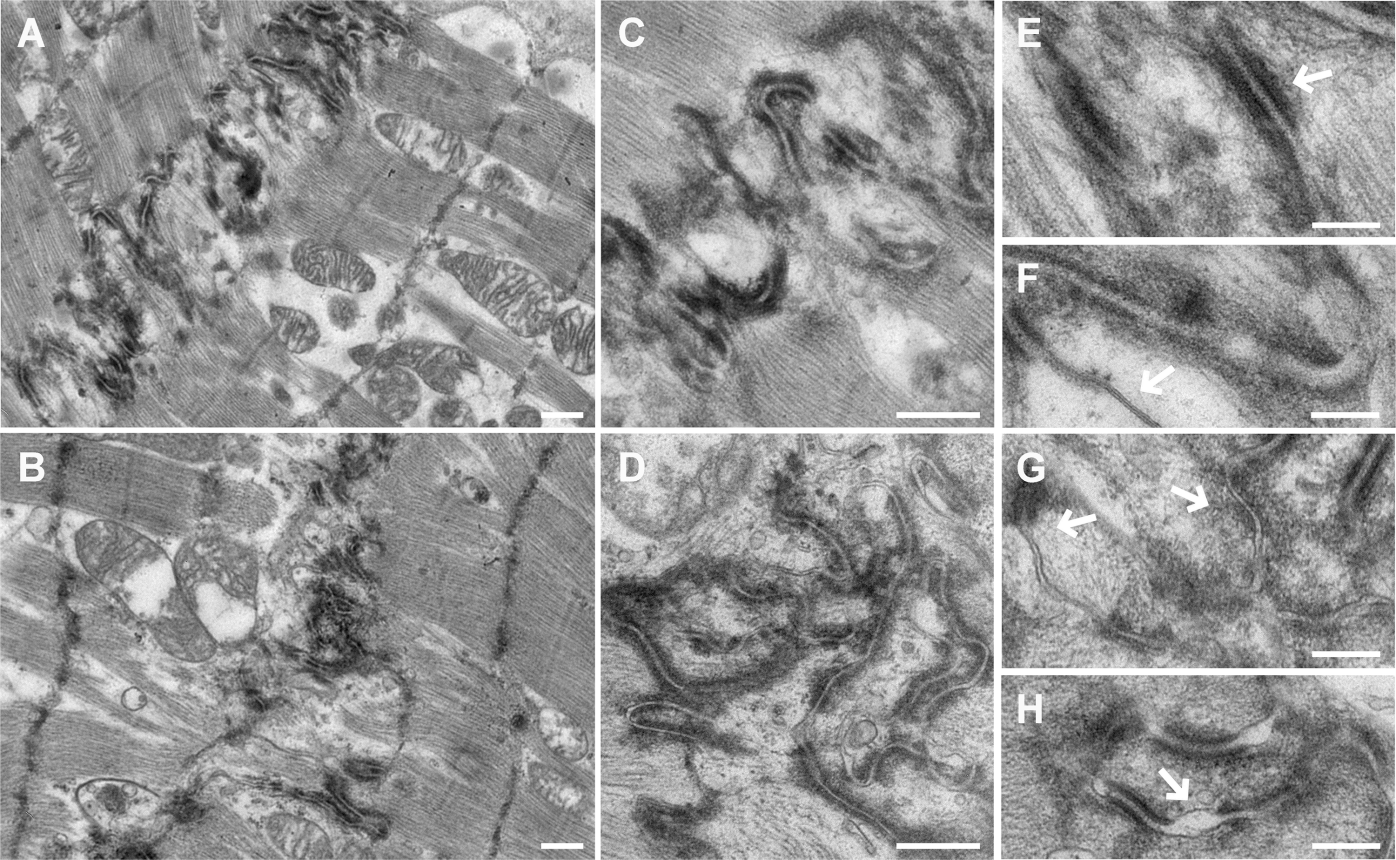
Transmission electron microscopy micrographs of the DCM patients compared to the CNT group showing the ID structure disorganization. **(A–B)** Panoramic view of the ID structure in a CNT and DCM subjects, respectively. When compared to CNT, the DCM ID appeared disrupted and fewer convolutions were observed in the DCM group (2.20 ± 0.16 *vs*. 3.54 ± 0.20; *P* < 0.0001). **(C–D)** Magnifications of the ID organization in a CNT and DCM subjects, respectively. **(E)** Detail of desmosome in a CNT subject. **(F)** Detail of GJ in a CNT subject. **(G)** Detail of some desmosomes with paleness of internal plaques in a DCM patient. **(H)** Detail of widening of AJ in a DCM patient. Scale bars: **(A–D)**, 500 nm; **(E–H)**, 200 nm. AJ, adherens junction; CNT, control; DCM, dilated cardiomyopathy; GJ, gap junction; ID, intercalated disc.

**Fig 6:**
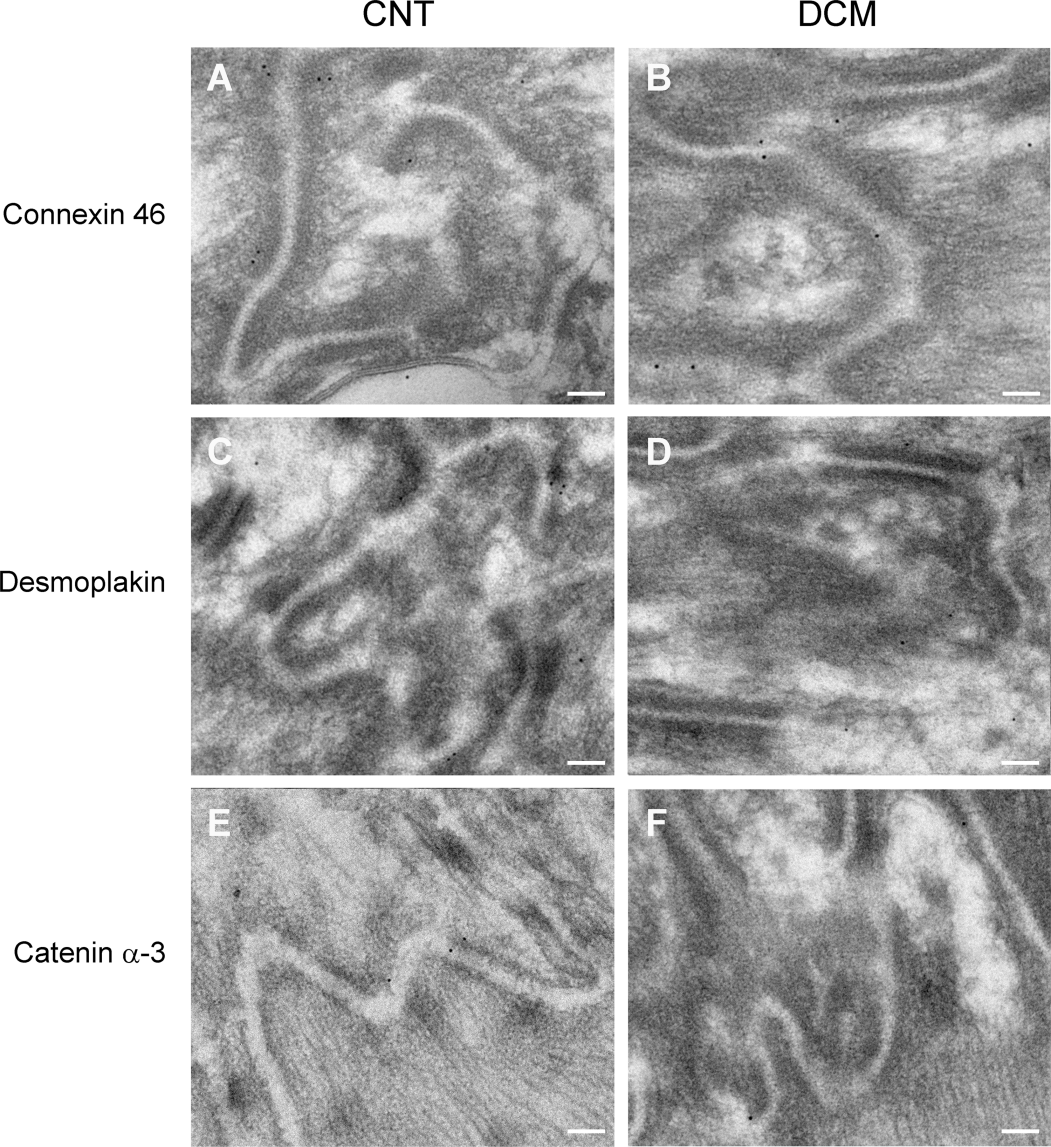
Immunohistochemistry of ID proteins displaying a reduction of immunogold labeling in samples from DCM patients compared to the CNT group. **(A–B)** Immunolabeling of connexin 46 in CNT and DCM samples, respectively. **(C–D)** Immunolabeling of desmoplakin in CNT and DCM samples, respectively. **(E–F)** Immunolabeling of catenin α-3 in CNT and DCM samples, respectively. Scale bar: 100 nm. CNT, control; DCM, dilated cardiomyopathy; ID: intercalated disc.

Additionally, we studied the ID structure in the DCM and CNT samples. The transmission electron microscopy observation showed ID structural differences between the groups (Fig 5), in which we observed disorganizations in DCM tissue, desmosome paleness and widening of AJ gaps. In the analysis, the ID length was evaluated according to the convolution index (the real distance of the ID divided by the end-to-end distance). The patients with DCM showed a lower convolution index when compared with that of the CNT (2.20 ± 0.16 vs. 3.54 ± 0.20; *P* < 0.0001). We also performed immunohistochemistry studies of connexin 46, desmoplakin and catenin α-3 proteins and we observed a reduction in immunogold labeling in DCM samples of the three proteins analyzed (Fig 6).

## Discussion

In this study, we demonstrated the existence of an altered transcriptomic profile of the cell adhesion machinery in patients with DCM. At the same time, the altered expression of the connexin, the desmosomal and the catenin genes studied, was directly related with the EF of the left ventricle and with the LV dimensions of these patients. RNA-sequencing analysis allowed us to examine the differences in gene expression between the groups in greater detail, focusing on cell adhesion related genes, category that has been highly enhanced by the functional enrichment analysis performed. We found 29 altered genes, mainly components of the ID structure, which expression pattern was clearly separated between the DCM and CNT groups, as evidenced by the Heat map and Hierarchical clustering analyses. We also performed gene expression validations by RT-qPCR and protein and immunohistochemistry analyses that were coincident with the gene expression alterations found in ID components.

In previous studies, we analyzed the differential expression of genes coding for cell adhesion molecules in ischemic cardiomyopathy, another major cause of HF, and identified molecules affecting this pathology and related with cardiac function different from the ones we have found in this study [25]. All these results together suggest the presence of distinct contributors affecting myocardial dysfunction in these two HF-triggering diseases. In this sense, we provide further evidence that the two etiologies follow different paths in the progression to HF.

Cardiac contractility is affected in HF. IDs are important specialized structures, unique to the heart muscle and responsible for the coupling of the contraction process [26]. This study shows differential expression of genes and proteins coding for the three main junctional constituents of the ID (GJ, desmosomes and AJ) and important correlations with the functional status of patients.

GJ are important in electrical and metabolic coupling between cardiac myocytes and for normal cardiac pump function [27]. We found that the genes *GJC1* (connexin 45) and *GJA3* (connexin 46) were differentially expressed in patients with DCM. Although the most expressed connexin in the heart muscle is connexin 43 (*GJA1* gene), which has shown reduced protein content in HF [28, 29], changes in its gene expression are controversial [30, 31]. In this study, we did not find significant mRNA level alterations, possibly because of modifications that occurred at the post-translational level. Conversely, other less studied genes, such as *GJA3*, (connexin 46) showed a down-regulation in gene and protein expression and relationships with EF and LV dimensions. *GJA3* has been implicated in cardiac conduction disturbances in animal models [32], but no studies have analyzed its expression at gene or protein level in HF syndrome. The link with LV dysfunction found in this analysis indicates an association with a worse functional status in patients, evidencing important roles for *GJA3* in HF physiopathology not previously analyzed. *GJC1* showed increased mRNA levels, according to some author’s studies at protein level [33]. All these alterations in connexin genes reinforce the notion of gap junctional remodeling in HF syndrome, as stated by several authors [29, 34] and evidence the crucial role of specific genes such as *GJA3*.

On the other hand, desmosomal genes *DSP* (desmoplakin) and *PKP4* (plakophilin) have shown a down-regulation in DCM subjects. Mutations in *DSP* have been found in patients with arrhythmogenic right ventricular cardiomyopathy and DCM [35, 36]. However, *PKP4* gene down-regulation or mutational alterations have not previously been described in HF syndrome. This finding may open novel paths to investigate other desmosomal genes involved in this pathology. We also lack studies reporting protein expression analysis of desmoplakin not associated to *DSP* mutations. In our study, this protein showed reduced levels in pathological samples, which is consistent with the quantification of mRNA levels. We found that the most abundant molecule of desmosomal structure, coded by the *DSP* gene, was related to EF and both LV diameters, showing that its decreased expression is accompanied by poor ventricular function in patients. Mice models, with specific loss-of-function genetic variants of *Dsp* have shown cardiac alterations, including wall thickness, increase in LV diameters and decreased EF [37], which supports the interesting findings of the relationships we have found in humans. These desmosomal components interact with key constituents of cytoskeleton, molecules that we have previously reported to be altered in patients with HF [38]. It is likely; therefore, that ventricular dysfunction occurs, at least in part, as a result of reduced cytoskeletal integrity and impaired cell adhesion machinery.

Catenins link the cadherin-based cell-cell adhesion complex to the cytoskeleton, to mediate cell-cell adhesion. This analysis shows that 31% of deregulated genes in DCM belong to the cadherin superfamily or cadherin-related genes, constituting the AJ. Interestingly, *CTNNA3* (encoding catenin α-3) showed diminished protein levels in DCM and its reduced gene expression was related to EF and LV dimensions, which is consistent with previous results where animal models with a loss-of-function of *CTNNA3* developed early DCM [39]. These earlier results and the correlations with the impaired LV function of patients found in this study, confirm the relevant role of this molecule in DCM progression.

Analyzing the relationships in cell adhesion genes related to LV function, we found that were also related between all of them. Some authors have shown a molecular link between the catenin α-3 (AJ) and desmosomes in the heart [8]. These specific links, found between genes belonging to the three main junction structures of the ID, and the direct relationships found with EF and LV dimensions, increases the relevance and evidences the presence of an interrelation of these ID molecules in the mechanisms involving LV perturbations in human DCM. Since these molecules participate actively and physically in the interaction that occur in the ID, that mechanically and electrically attach cardiomyocytes to each other enabling the contraction process, their alterations, and above, their down-regulation may be influencing the ventricular function decay observed in these patients.

We further examined the images of the human LV samples obtained using electron microscopy, which showed increased breakdown of the ID and desmosomes in subjects with DCM. Indeed, we observed widening of AJ gaps in patients, as previously observed by Basso et al. in arrhythmogenic right ventricular cardiomyopathy patients [24]. In addition, the ID convolution index is diminished in these patients, and other alterations such as paleness of desmosomal internal plaques and gaps between cells were found in the DCM but not in the CNT samples, an observation not previously reported by other authors in human tissue. We complemented the ultrastructure observations with immunohistochemistry studies of connexin 46, desmoplakin and catenin α-3 which showed reduced immunolabeling in DCM samples. These results are consistent with the changes observed in the expression of ID genes and proteins, evidencing a crucial role for these molecules in ID structure organization.

A common limitation of the studies using samples from end-stage failing human hearts is the fact that there is a high variability in disease etiology and patients are under heavy medical treatment.

In conclusion, this study shows novel expression alterations in cell adhesion genes and proteins of patients with DCM. Furthermore, three of these genes, all components of the microscopically altered ID structure, *GJA3*, *DSP* and *CTNNA3*, have shown relevant relationships with the depressed LV functional status and remodeling. These findings give new data for understanding the ventricular depression that characterizes DCM, opening a new therapeutic perspective for these critically diseased patients.

## Supporting information

S1 Table Cell adhesion category genes in DCM patients. Table shows gene expression levels and p-values of cell adhesion genes in DCM patients compared to CNT subjects CNT, control; DCM, dilated cardiomyopathy; FC, fold change.
(XLSX)

## References

[1] Pinto YM, Elliott PM, Arbustini E, Adler Y, Anastasakis A, Bohm M, et al Proposal for a revised definition of dilated cardiomyopathy, hypokinetic non-dilated cardiomyopathy, and its implications for clinical practice: a position statement of the ESC working group on myocardial and pericardial diseases. Eur Heart J. 2016;37: 1850–1858. doi: 10.1093/eurheartj/ehv727 26792875 10.1093/eurheartj/ehv727

[2] Jefferies JL, Towbin JA. Dilated cardiomyopathy. Lancet. 2010;375: 752–762. doi: 10.1016/S0140-6736(09)62023-7 20189027 10.1016/S0140-6736(09)62023-7

[3] Ortega A, Rosello-Lleti E, Tarazon E, Molina-Navarro MM, Martinez-Dolz L, Gonzalez-Juanatey JR, et al Endoplasmic reticulum stress induces different molecular structural alterations in human dilated and ischemic cardiomyopathy. PLoS One. 2014;9: e107635 doi: 10.1371/journal.pone.0107635 25226522 10.1371/journal.pone.0107635 PMC4166610

[4] Rosello-Lleti E, Tarazon E, Barderas MG, Ortega A, Molina-Navarro MM, Martinez A, et al ATP synthase subunit alpha and LV mass in ischaemic human hearts. J Cell Mol Med. 2015;19: 442–451. doi: 10.1111/jcmm.12477 25382018 10.1111/jcmm.12477 PMC4407605

[5] Wilson AJ, Schoenauer R, Ehler E, Agarkova I, Bennett PM. Cardiomyocyte growth and sarcomerogenesis at the intercalated disc. Cell Mol Life Sci. 2014;71: 165–181. doi: 10.1007/s00018-013-1374-5 23708682 10.1007/s00018-013-1374-5 PMC3889684

[6] Vermij SH, Abriel H, van Veen TA. Refining the molecular organization of the cardiac intercalated disc. Cardiovasc Res. 2017;113: 259–275. doi: 10.1093/cvr/cvw259 28069669 10.1093/cvr/cvw259

[7] Garcia-Pavia P, Syrris P, Salas C, Evans A, Mirelis JG, Cobo-Marcos M, et al Desmosomal protein gene mutations in patients with idiopathic dilated cardiomyopathy undergoing cardiac transplantation: a clinicopathological study. Heart. 2011;97: 1744–1752. doi: 10.1136/hrt.2011.227967 21859740 10.1136/hrt.2011.227967

[8] Goossens S, Janssens B, Bonne S, De Rycke R, Braet F, van Hengel J, et al A unique and specific interaction between alphaT-catenin and plakophilin-2 in the area composita, the mixed-type junctional structure of cardiac intercalated discs. J Cell Sci. 2007;120: 2126–2136. doi: 10.1242/jcs.004713 17535849 10.1242/jcs.004713

[9] Janssens B, Mohapatra B, Vatta M, Goossens S, Vanpoucke G, Kools P, et al Assessment of the CTNNA3 gene encoding human alpha T-catenin regarding its involvement in dilated cardiomyopathy. Hum Genet. 2003;112: 227–236. doi: 10.1007/s00439-002-0857-5 12596047 10.1007/s00439-002-0857-5

[10] Sohl G, Willecke K. Gap junctions and the connexin protein family. Cardiovasc Res. 2004;62: 228–232. doi: 10.1016/j.cardiores.2003.11.013 15094343 10.1016/j.cardiores.2003.11.013

[11] Hesketh GG, Shah MH, Halperin VL, Cooke CA, Akar FG, Yen TE, et al Ultrastructure and regulation of lateralized connexin43 in the failing heart. Circ Res. 2010;106: 1153–1163. doi: 10.1161/CIRCRESAHA.108.182147 20167932 10.1161/CIRCRESAHA.108.182147 PMC2896878

[12] Morimoto S. Sarcomeric proteins and inherited cardiomyopathies. Cardiovasc Res. 2008;77: 659–666. doi: 10.1093/cvr/cvm084 18056765 10.1093/cvr/cvm084

[13] McMurray JJ, Adamopoulos S, Anker SD, Auricchio A, Bohm M, Dickstein K, et al ESC Guidelines for the diagnosis and treatment of acute and chronic heart failure 2012: The Task Force for the Diagnosis and Treatment of Acute and Chronic Heart Failure 2012 of the European Society of Cardiology. Developed in collaboration with the Heart Failure Association (HFA) of the ESC. Eur Heart J. 2012;33: 1787–1847. doi: 10.1093/eurheartj/ehs104 22611136 10.1093/eurheartj/ehs104

[14] Macrae DJ. The Council for International Organizations and Medical Sciences (CIOMS) guidelines on ethics of clinical trials. Proc Am Thorac Soc. 2007;4: 176–178, discussion 178–179. doi: 10.1513/pats.200701-011GC 17494727 10.1513/pats.200701-011GC

[15] Li H, Handsaker B, Wysoker A, Fennell T, Ruan J, Homer N, et al The Sequence Alignment/Map format and SAMtools. Bioinformatics. 2009;25: 2078–2079. doi: 10.1093/bioinformatics/btp352 19505943 10.1093/bioinformatics/btp352 PMC2723002

[16] Trapnell C, Williams BA, Pertea G, Mortazavi A, Kwan G, van Baren MJ, et al Transcript assembly and quantification by RNA-Seq reveals unannotated transcripts and isoform switching during cell differentiation. Nat Biotechnol. 2010;28: 511–515. doi: 10.1038/nbt.1621 20436464 10.1038/nbt.1621 PMC3146043

[17] Anders S, Pyl PT, Huber W. HTSeq—a Python framework to work with high-throughput sequencing data. Bioinformatics. 2015;31: 166–169. doi: 10.1093/bioinformatics/btu638 25260700 10.1093/bioinformatics/btu638 PMC4287950

[18] Robinson MD, McCarthy DJ, Smyth GK. edgeR: a Bioconductor package for differential expression analysis of digital gene expression data. Bioinformatics. 2010;26: 139–140. doi: 10.1093/bioinformatics/btp616 19910308 10.1093/bioinformatics/btp616 PMC2796818

[19] Edgar R, Domrachev M, Lash AE. Gene Expression Omnibus: NCBI gene expression and hybridization array data repository. Nucleic Acids Res. 2002;30: 207–210. 11752295 10.1093/nar/30.1.207 PMC99122

[20] Livak KJ, Schmittgen TD. Analysis of relative gene expression data using real-time quantitative PCR and the 2(-Delta Delta C(T)) Method. Methods. 2001;25: 402–408. doi: 10.1006/meth.2001.1262 11846609 10.1006/meth.2001.1262

[21] Chen J, Bardes EE, Aronow BJ, Jegga AG. ToppGene Suite for gene list enrichment analysis and candidate gene prioritization. Nucleic Acids Res. 2009;37: W305–311. doi: 10.1093/nar/gkp427 19465376 10.1093/nar/gkp427 PMC2703978

[22] Tomas M, Fornas E, Megias L, Duran JM, Portoles M, Guerri C, et al Ethanol impairs monosaccharide uptake and glycosylation in cultured rat astrocytes. J Neurochem. 2002;83: 601–612. 12390522 10.1046/j.1471-4159.2002.01167.x

[23] Lucocq J. Quantification of structures and gold labeling in transmission electron microscopy. Methods Cell Biol. 2008;88: 59–82. doi: 10.1016/S0091-679X(08)00404-4 18617028 10.1016/S0091-679X(08)00404-4

[24] Basso C, Czarnowska E, Della Barbera M, Bauce B, Beffagna G, Wlodarska EK, et al Ultrastructural evidence of intercalated disc remodelling in arrhythmogenic right ventricular cardiomyopathy: an electron microscopy investigation on endomyocardial biopsies. Eur Heart J. 2006;27: 1847–1854. doi: 10.1093/eurheartj/ehl095 16774985 10.1093/eurheartj/ehl095

[25] Ortega A, Gil-Cayuela C, Tarazon E, Garcia-Manzanares M, Montero JA, Cinca J, et al New Cell Adhesion Molecules in Human Ischemic Cardiomyopathy. PCDHGA3 Implications in Decreased Stroke Volume and Ventricular Dysfunction. PLoS One. 2016;11: e0160168 doi: 10.1371/journal.pone.0160168 27472518 10.1371/journal.pone.0160168 PMC4966940

[26] Fry CH, Gray RP, Dhillon PS, Jabr RI, Dupont E, Patel PM, et al Architectural correlates of myocardial conduction: changes to the topography of cellular coupling, intracellular conductance, and action potential propagation with hypertrophy in Guinea-pig ventricular myocardium. Circ Arrhythm Electrophysiol. 2014;7: 1198–1204. doi: 10.1161/CIRCEP.114.001471 25313260 10.1161/CIRCEP.114.001471

[27] Kanno S, Saffitz JE. The role of myocardial gap junctions in electrical conduction and arrhythmogenesis. Cardiovasc Pathol. 2001;10: 169–177. 11600334 10.1016/s1054-8807(01)00078-3

[28] Wang X, Gerdes AM. Chronic pressure overload cardiac hypertrophy and failure in guinea pigs: III. Intercalated disc remodeling. J Mol Cell Cardiol. 1999;31: 333–343. doi: 10.1006/jmcc.1998.0886 10093046 10.1006/jmcc.1998.0886

[29] Severs NJ. Gap junction remodeling in heart failure. J Card Fail. 2002;8: S293–299. doi: 10.1054/jcaf.2002.129255 12555135 10.1054/jcaf.2002.129255

[30] Dupont E, Matsushita T, Kaba RA, Vozzi C, Coppen SR, Khan N, et al Altered connexin expression in human congestive heart failure. J Mol Cell Cardiol. 2001;33: 359–371. doi: 10.1006/jmcc.2000.1308 11162139 10.1006/jmcc.2000.1308

[31] Soltysinska E, Olesen SP, Christ T, Wettwer E, Varro A, Grunnet M, et al Transmural expression of ion channels and transporters in human nondiseased and end-stage failing hearts. Pflugers Arch. 2009;459: 11–23. doi: 10.1007/s00424-009-0718-3 19768467 10.1007/s00424-009-0718-3

[32] Chi NC, Bussen M, Brand-Arzamendi K, Ding C, Olgin JE, Shaw RM, et al Cardiac conduction is required to preserve cardiac chamber morphology. Proc Natl Acad Sci U S A. 2010;107: 14662–14667. doi: 10.1073/pnas.0909432107 20675583 10.1073/pnas.0909432107 PMC2930423

[33] Yamada KA, Rogers JG, Sundset R, Steinberg TH, Saffitz J. Up-regulation of connexin45 in heart failure. J Cardiovasc Electrophysiol. 2003;14: 1205–1212. 14678136 10.1046/j.1540-8167.2003.03276.x

[34] Bruce AF, Rothery S, Dupont E, Severs NJ. Gap junction remodelling in human heart failure is associated with increased interaction of connexin43 with ZO-1. Cardiovasc Res. 2008;77: 757–765. doi: 10.1093/cvr/cvm083 18056766 10.1093/cvr/cvm083 PMC5436744

[35] Bauce B, Basso C, Rampazzo A, Beffagna G, Daliento L, Frigo G, et al Clinical profile of four families with arrhythmogenic right ventricular cardiomyopathy caused by dominant desmoplakin mutations. Eur Heart J. 2005;26: 1666–1675. doi: 10.1093/eurheartj/ehi341 15941723 10.1093/eurheartj/ehi341

[36] Norgett EE, Hatsell SJ, Carvajal-Huerta L, Cabezas JC, Common J, Purkis PE, et al Recessive mutation in desmoplakin disrupts desmoplakin-intermediate filament interactions and causes dilated cardiomyopathy, woolly hair and keratoderma. Hum Mol Genet. 2000;9: 2761–2766. 11063735 10.1093/hmg/9.18.2761

[37] Yang Z, Bowles NE, Scherer SE, Taylor MD, Kearney DL, Ge S, et al Desmosomal dysfunction due to mutations in desmoplakin causes arrhythmogenic right ventricular dysplasia/cardiomyopathy. Circ Res. 2006;99: 646–655. doi: 10.1161/01.RES.0000241482.19382.c6 16917092 10.1161/01.RES.0000241482.19382.c6

[38] Herrer I, Rosello-Lleti E, Rivera M, Molina-Navarro MM, Tarazon E, Ortega A, et al RNA-sequencing analysis reveals new alterations in cardiomyocyte cytoskeletal genes in patients with heart failure. Lab Invest. 2014;94: 645–653. doi: 10.1038/labinvest.2014.54 24709777 10.1038/labinvest.2014.54

[39] Li J, Goossens S, van Hengel J, Gao E, Cheng L, Tyberghein K, et al Loss of alphaT-catenin alters the hybrid adhering junctions in the heart and leads to dilated cardiomyopathy and ventricular arrhythmia following acute ischemia. J Cell Sci. 2012;125: 1058–1067. doi: 10.1242/jcs.098640 22421363 10.1242/jcs.098640 PMC3311935

